# Retrotransposons in Plant Genomes: Structure, Identification, and Classification through Bioinformatics and Machine Learning

**DOI:** 10.3390/ijms20153837

**Published:** 2019-08-06

**Authors:** Simon Orozco-Arias, Gustavo Isaza, Romain Guyot

**Affiliations:** 1 Department of Computer Science, Universidad Autónoma de Manizales, Manizales 170001, Colombia; 2 Department of Systems and Informatics, Universidad de Caldas, Manizales 170001, Colombia; 3 Department of Electronics and Automatization, Universidad Autónoma de Manizales, Manizales 170001, Colombia; 4 Institut de Recherche pour le Développement, CIRAD, University Montpellier, 34000 Montpellier, France

**Keywords:** transposable elements, retrotransposons, function, structure, detection, classification, bioinformatics, machine learning, deep learning

## Abstract

Transposable elements (TEs) are genomic units able to move within the genome of virtually all organisms. Due to their natural repetitive numbers and their high structural diversity, the identification and classification of TEs remain a challenge in sequenced genomes. Although TEs were initially regarded as “junk DNA”, it has been demonstrated that they play key roles in chromosome structures, gene expression, and regulation, as well as adaptation and evolution. A highly reliable annotation of these elements is, therefore, crucial to better understand genome functions and their evolution. To date, much bioinformatics software has been developed to address TE detection and classification processes, but many problematic aspects remain, such as the reliability, precision, and speed of the analyses. Machine learning and deep learning are algorithms that can make automatic predictions and decisions in a wide variety of scientific applications. They have been tested in bioinformatics and, more specifically for TEs, classification with encouraging results. In this review, we will discuss important aspects of TEs, such as their structure, importance in the evolution and architecture of the host, and their current classifications and nomenclatures. We will also address current methods and their limitations in identifying and classifying TEs.

## 1. Introduction

Transposable elements (TEs) are genomic units able to move within and among the genomes of virtually all organisms [1]. They are the main contributors to genomic diversity and genome size variation [2], with the exception of polyploidy events. An important issue in genome sequence analyses is to rapidly identify and reliably annotate TEs. There are major obstacles and challenges in the analysis of these elements [3], including their repetitive nature, structural polymorphism, species specificity, and, conversely, their conservation across genera and families, as well as their high divergence rate, even across close relative species [4].

Among eukaryotic genomes, TEs represent the most repetitive sequences [5]. They are able to move in the genomes, generate mutations, and obviously amplify the number of their copies [6]. Usually they are classified according to their coding regions involved in the replication of the element [7]. TEs moving via an RNA molecule called retrotransposons fall into Class I, while elements moving via a DNA molecule, called transposons, are classified into Class II [8]. They represent the vast majority of TEs found in plant genomes due to their mobility mechanisms. Retrotransposons can be further subclassified into four orders according to their structural features and the element’s life cycle: Long Terminal Repeat retrotransposon (LTR-RT), non-LTR retrotransposons, PLEs, and DIRS (see Section 2).

LTR-RT is the most common order [9,10], and they can contribute up to 80% of the plant genome size, as in wheat, barley, or the rubber tree [11]. The LTR-RT order is composed of two superfamilies in plants: Copia and Gypsy, based on the internal organization of the coding domain [12]. Each Copia and Gypsy superfamily is further sub-classified into lineages and families [8] through phylogenetic analysis based on coding region similarities (often of the enzymatic domain known as reverse transcriptase) [13]. For plant genomes, Ale (also known as Retrofit), Alesia, Angela, Bianca, Bryco, Lyco, Gymco, Ikeros (also known as Tork sto-4), Ivana (or Oryco), Osser, SIRE, Tar (also known as Tork), and Tork lineages belong to the Copia superfamily, while Athila, Clamyvir, Galadriel, Selgy, Tcn1, Reina, Tekay (or Del), CRM (also named Centromeric Retrotransposon), Phygy, and TAT are grouped into the Gypsy superfamily [13,14]. Phylogenetic studies have divided Gypsy into different groups according to the presence of a chromodomain. The Galadriel, Reina, Tekay (Del), and CRM lineages were grouped into the Chromovirus branch [15,16].

Several methods were developed to identify and annotate transposable elements in sequenced genomes. These are classified into four categories: de novo, structure-based, comparative genomics, and homology-based [17]. These approaches offer different specificities and sensibilities and all suffer from a relatively high rate of false positive detections. Other methods based on the assembly of repetitive reads have been reported, such as RED [18], TEdna [19], Transposome [20], and REP denovo [21]. LTRClassifier [22], and Inpactor [23] were only dedicated for classification.

Machine learning (ML) is defined as algorithms that are able to improve and optimize a performance criterion based on already processed data or a past experience [24] to build a model. ML has been applied to many bioinformatics problems, including genomics [25], systems biology, and evolution [24], demonstrating substantial benefits in terms of precision and speed. Several recent studies using ML for the detection of TEs report drastic improvements of the results [26,27,28].

In this paper, we review the importance of transposable elements in genome architecture and evolution, as well as the need and challenge for a rapid and accurate detection and classification of TEs in an era of massive plant genome sequencing projects (such as the 10 K plant genomes project https://db.cngb.org/10kp). Finally, we discuss current bioinformatic methods and algorithms to detect and classify TEs, focusing on retrotransposons, as well as the state of the art of ML and Deep Learning approaches applied to TE fields.

## 2. Structure, Diversity, Dynamics, and Function of Retrotransposons in Host Genomes

Transposable elements (TEs) were first discovered by Barbara McClintock while she was experimenting on maize in 1944 [29]. Currently, it is well known that these elements cover a large portion of eukaryote genomes and play an important role within them [30]. LTR-RTs are the most abundant repeat element since they proliferate through an RNA-mediated copy-and-paste mechanism, rapidly increasing their copy number [31,32]. For instance, in maize and sugarcane, they account for approximately 40%–75% of the genomes [33]. LTR-RTs are also known for their variability in structures, functions, and locations inside genomes. For the reasons mentioned above, we focused on them in this review.

### 2.1. Retrotransposons Structure

Retrotransposons or Class I are commonly divided into four orders following Wicker’s classification (with the exception of the LINEs and SINEs that compose the Order non-LTR retrotransposons): LTR retrotransposons, non-LTR retrotransposons, PLEs, and DIRS [34]. All of these have significant differences in their structure, the presence and organization of enzymatic domains, motifs or regulatory regions, and in their life cycle [35].

#### 2.1.1. LTR Retrotransposons

The structural organization of LTR-RTs is similar to that of retroviruses [9,36] except for the absence or non-functional presence of the envelope (env) gene [37,38]. LTR-RTs are extremely variable in size, ranging in plants from 4 kb to over 31–23 kb [39,40] (i.e., Ogre elements with >23 kb in length [41,42]) for functional and complete elements [12,42,43]. A key feature in this order is the presence of long terminal repeats (LTRs), which are two homologous (identical at the time of insertion) non-coding DNA sequences [44] located at both ends of the internal coding region and can range from a few hundred base pairs to more than 5 kb [26]. LTR-RTs contain one [45] or more open reading frames (ORF) [46] that are transcribed using host machinery [47] and code for *gag* and *polyprotein* (*pol*) genes. They can be separated by one or more stop codons. *Gag* genes are generally the most variable LTR retrotransposon domains, even if they encode a major structural protein [37,48], and are responsible for the packaging of retrotransposon RNA and proteins [49]. The *polyprotein* gene encodes some enzymatic domains such as the *aspartic proteinase* (*AP*), *reverse transcriptase* (*RT*), *RNase H* [50], and *integrase* (*INT*) [51,52,53]. Each domain has a specific role in the replication cycle [54] (Table 1). In some cases, they have another region upstream the 3′ LTR called chromodomain that can be responsible for targeted integration [38,52,55] and for escaping silencing by the specific targeting of heterochromatic regions [55].

Some plant LTR retrotransposons, like Sire [56], can also contain an extra ORF encoding a domain usually named “*EN*V-*like*” (envelope-like), which is analogous of the envelope gene required for infection in a retrovirus. A similar function has not been clearly demonstrated for LTR-RTs [9]. Regulation of the excess production of *gag* formation is a critical process in the retrotransposon life cycle because it requires higher expression levels of the *group specific antigen* (*gag*) protein compared to other enzymatic components [57].

Long terminal repeat (LTR) sequences are non-coding regions evolving more rapidly than other components of LTR-RTs [58]. They contain start and stop signals of transcription [53,57,59], polyadenylation signals, and enhancers [60] that are critical to the replication process [10]. LTRs are generally composed of U3, R, and U5 domains [10,61], each one with a specific function in the retrotranscription process [62]. R and U5 sections are generally more conservative than U3, probably due to the adaptation to varying tissue environments [63] and to different stress responses [34]. Interestingly, LTRs of retrotransposon and retroviruses share comparable function in the initiation of the RNA template, the first step of the movement of the element [37]. Since the RNA template is generated from R to R sections, it contains only one U5 and U3 section, and eventually, two identical LTRs when the DNA copy of the element is inserted into the genome [64]. A short motif TG-5′ and 3′-CA called the Short Inverted Repeat (SIR) initiates and terminates LTRs [65,66]. However, some exception to these conserved motifs were reported in Rosaceae species [67]. Besides the presence of one or two TATA-boxes and a polyadenylation signal (AATAAA motif), they are generally composed of AT-rich regions [10,63].

**Figure 1:**
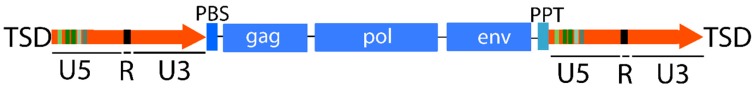
Structure of LTR retrotransposon. The *env* gene might not be present in some elements. Orange arrows correspond to LTRs.

LTR-RTs also contain a primer binding site (PBS) and a Poly-Purine Tract (PPT). Both sites can work as primers [64], whereby the first is the (−)-strand priming site for reverse transcription and the second is the (+)-strand priming site for reverse transcription [31,46,68]. In addition, the neo-insertion of LTR retrotransposons creates a short duplication called the target site duplication (TSD) of 4–6 bp at the termini of the element [12,40,69,70] (Figure 1).

**Figure 2:**
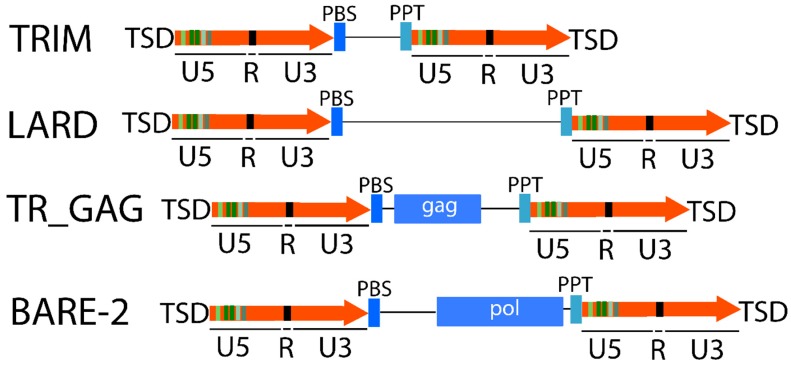
Structure of non-autonomous elements. Orange arrows correspond to LTRs and single lines correspond to non-coding regions. PBS: primer binding site; PPT: Poly-Purine Tract; TRIM: Terminal-Repeat Retrotransposons in Miniature; LARD: LArge Retrotransposon Derivatives.

Several LTR-RTs are present in very high copy numbers in many genomes, but most of them lack the functional genes necessary for transposition. Some of them can parasitize the functional machinery produced by other LTR-RTs to retrotranspose [7,12,65,71]. These elements are called non-autonomous [62] and are classified according to their structures into Terminal-Repeat Retrotransposons in Miniature (TRIM) [72], which are very small in size (from a few hundred bases to 4 kbp [40,43,73]), LARD (of length greater than 4 kb) [74], TR_GAG [7], and BARE-2 (Figure 2).

#### 2.1.2. Non-LTR Retrotransposons

**Figure 3:**
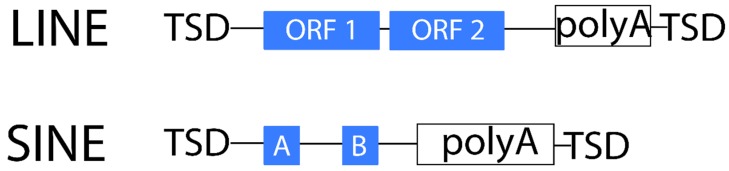
Structure of non-LTR retrotransposons.

Non-LTR retrotransposons lack LTRs and are transcribed from an internal promoter. These elements can replicate without an INT domain. Instead, the RT domain initiates DNA synthesis from the poly-A tail of the non-LTR retrotransposon transcript and, finally, ligates the end of the newly synthesized DNA into the insertion point [75]. These elements are generally much less abundant in plants than LTR retrotransposons [75]. They are usually sub-classified into long interspersed nuclear elements or LINEs and short interspersed nuclear elements or SINEs. Similar to LTR retrotransposons, LINEs have *gag* and *pol* coding regions, which encode domains that play important roles in structural and enzymatic activities [62]. As in the LTR-RT life cycle, SINE elements lack the ability to self-replicate (non-autonomous) and thus depend on the LINE mechanism [31,76]. SINEs are composed of various tRNA, rRNA, and other polymerase III transcripts ranging from 75 to 662 bp [31]. In contrast, LINEs generally encode *reverse transcriptase* and *endonuclease* genes within the same ORF and are thought to be transcribed by the RNA polymerase II, reaching several kbp in length [76] (Figure 3).

Non-LTR retrotransposons often contain a poly-A tail at the 3′end as a result of the transcription cycle [58,77]. SINEs are also terminated by an A-rich tail but, unlike LINEs, they have a sequence similarity to the host genes. Similar to LTR retrotransposons, LINEs and SINEs produce TSDs, yet non-LTR retrotransposons create TSDs of variable size on the insertion site [78].

#### 2.1.3. PLEs or Penelope-Like Elements

**Figure 4:**
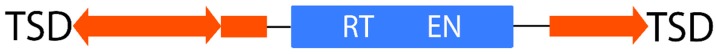
Structure of Penelope-like elements (PLEs).

PLEs are widely distributed from amoebae and fungi to vertebrates, but not in mammals. Very few of them have been detected in plants so far (Conifers). PLEs are composed of a single ORF that codes for some domains, including the *reverse transcriptase* (*RT*) and *endonuclease* (*EN*) [29] (Figure 4). Interestingly, the RT domain more closely resembles a telomerase than the RT from other retrotransposons such as LTR retrotransposons or LINEs. The EN domain is related to GIY-YIG intron encoded *endonucleases*. Some PLE elements also have sequences similar to LTR but can be oriented in a direct or inverse manner and have a functional intron [29]. Like LTR and non-LTR retrotransposons, PLEs produce TSD, but with a variable length. Interestingly, the integration mechanism of PLEs remains uncertain [79].

#### 2.1.4. DIRS

**Figure 5:**
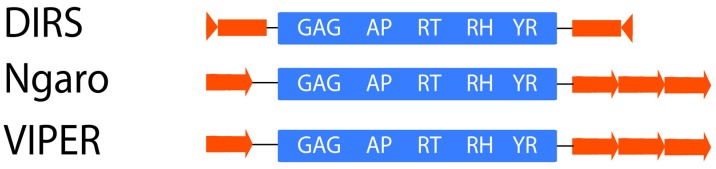
Structure of Dictyostelium intermediate repeat sequences (DIRS).

The DIRS (Dictyostelium intermediate repeat sequence [33]) order represents a structurally diverse group of retrotransposons that contain a *tyrosine recombinase* (YR) gene instead of an *INT* [79] and do not produce TSDs (Figure 5). The endings are similar to split direct repeats (SDR) or inverted repeats. These characteristics suggest an integration mechanism different from that of other retrotransposons. DIRSs are present in virtually all organisms, including plants [29]. They can be further classified into superfamilies like DIRS, Ngaro, and VIPER [8].

### 2.2. Retrotransposon Dynamics

Recent evidence has demonstrated that host genomes are able to regulate retrotransposon mobilization, resulting in extremely variable activities across different taxa and species [76]. Likewise, there are time periods when TEs are very active and when only a few (or no) new TE insertions occur [9,80,81]. A large number of residual TEs sequences prove that genomes also have efficient post-insertion mechanisms of TE removal and inactivation [62]. In the specific case of LTR-RTs, the LTR sequences are strictly identical when an LTR-RT is inserted. Thus, its insertion time can be calculated by the sequence divergence of two LTRs through an appropriate mutation rate [66]. This calculation is important to estimate the evolutionary dynamics of each type of retrotransposon in the host.

#### 2.2.1. How are Retrotransposons Activated

A highly dynamic genome is important for sessile organisms such as plants [35], and this may explain why the activation of TEs in plants is produced by internal or external elicitors [33,82,83]. There are multiple stresses acting on plants, including biotic and abiotic stresses, such as pathogens [84], pathogen elicitors [85], defense-associated stresses [62], tissue culture, wounding, heat, drought and salt stresses, freezing, polyploidization and hybridization events [86,87], UV light [75], and X-ray irradiation [9,57]. Although the activation of retrotransposons is a well-known phenomenon, in certain cases, the stress-induced retrotransposon response can be genotype-specific [33].

In LTR-RTs, the response to external stresses is attributed to the LTR sequences located at both ends [88]. On the other hand, activation of TEs is not always directly produced by external stresses but by the effect of those stresses on other cellular mechanisms that allow a rapid activation of some specific families of LTR retrotransposons [34]. In addition, some reports suggest that plant retrotransposons can escape host silencing by expressing anti-silencing factors [82]. Although retrotransposons are able to invade and densely populate plant genomes, only a few transpositionally active TEs have been identified and isolated so far in plants [89]. Table 2 shows several examples of stress-activated retrotransposons reported in plant genomes.

#### 2.2.2. How Are Retrotransposons Silenced

In order to prevent potential deleterious actions by retrotransposons [60,106], the host applies strategies to keep TE activities under control. Thus, in most plant genomes, the majority of intact LTR-RTs are recently inserted [82], while the others are found inactivated. Under normal conditions most of the plant retrotransposons are transcriptionally inactive [67,81,107,108]. For example, plants have evolved to reinforce certain processes of inactivation of retrotransposons in germline cells [109].

Different mechanisms of silencing were reported such as small interfering RNAs (siRNAs) via the production of TE double-stranded RNAs (dsRNAs) [49] involved in transcriptional silencing via DNA methylation and chromatin modification and in post-transcriptional silencing via degradation of TE mRNA (discussed in [12]), epigenetic mechanisms [82,110], activity inhibited by methylation [59,90,111], and histone modification [112], among others.

On the other hand, host genomes employ a variety of genome downsizing strategies to mitigate genome expansion caused by TEs [51]. For instance, one strategy is unequal recombination between LTRs of the same or different retrotransposons [49,64,82], which produces solo-LTRs in a single step by deleting one LTR and the internal section of them. Another strategy includes illegitimate recombination that gradually eliminates tracts of LTR retrotransposons and leaves truncated LTR retrotransposons [113,114]. Additionally, TEs seem to be under purifying selection [115], such as the direct disruptive effects of insertions, deleterious TE product expression, or chromosomal aberrations arising from ectopic recombination among TEs [113].

#### 2.2.3. Horizontal Transfer of TEs

Unlike the transmission of genetic material through the reproduction of living organisms, horizontal transfer (HT) is the process of exchanging genetic information using other methods [67], for example, vectors (bacteria or insects). There is evidence that HT events involve TEs (HTT) [116,117] in plant genomes. For example, Sharma and Presting [118] reported the HT of LTR retrotransposons between the Panicoid and Oryzoid lineages. El Baidouri et al. [119] demonstrated HTTs between at least 26 plant species. Hou et al. [120] found HTT events among seven Rosales species. Dias et al. [116] hypothesized the HTT of an LTR retrotransposon called “Copia25” between the ancestors of the genera *Ixora* and *Musa*. Although the mechanisms of HTT remain unclear in plants, HTT represents an important way for host genomes to innovate and evolve [120].

### 2.3. Function of Retrotransposons in a Chromosome’s Structure

Initially, transposable elements were attributed to negative effects in the host genomes [29], but in recent years, several studies have demonstrated key roles [121], such as reorganization of the genome after polyploidization events [122], promotion of male gene expression in late spermatogenesis [123], chromosome organization (in particular, at sexual chromosomes), involvement in rearrangement events [78,82] (e.g., translocations, fusions or fissions), and contribution to genome size variations [124].

#### 2.3.1. Chromosomal Distribution of Retrotransposons

Chromatin is composed of heterochromatin, which is densely compacted during most of the cell cycle, and euchromatin, with a relatively less dense organization [125]. Heterochromatin is visualized through staining pachytene chromosomes during the interphase of cell division and is important for meiotic chromosome segregation [126]. Heterochromatin can be divided into two types according to its components: constitutive, which is mainly composed of repetitive elements, and facultative, which is found in gene-rich portions [126]. 

Although TEs are more frequent in heterochromatin [83], each LTR retrotransposon superfamily shows distinct chromosomal distribution patterns [12,43] supported by FISH experiments. In plants, Copia were found to be mainly distributed along the chromosomes with a preference for euchromatin [82,127], where their presence may act as key factors in chromosome rearrangements, gene gain, and loss, as well as epigenetic marks [128]. In contrast, Gypsy retrotransposons were found in heterochromatin where they serve as key components maintaining chromosome stability and heterochromatic silencing [70,129]. Similar to Gypsy, LINEs show a distribution along centromeric and/or pericentromeric regions [124]. 

In pericentromeric heterochromatic regions, recombinations are less frequent than in other chromosomal sections, creating different patterns of evolution between orthologous genes of two species. Thus, long pericentromeric regions with a high portion of TEs add chromosomal compartments with some evolutionary restrictions, which may be very suitable for several types of genes [82]. The observed distribution pattern of LTR retrotransposon families might result from the evolution of the inserted regions rather than insertional preferences. Insertions in pericentromeric regions could produce fewer mutations than in gene-rich regions, and genetic recombination in these regions is often completely suppressed. Instead, insertions in gene-rich regions can be severely counter-selected by evolution [130], leading to an apparent suppression of the insertion. 

A specific region of the chromosome, called centromere, plays a crucial function in chromosome segregation during cell division [131,132,133] and is critical for the differentiation of subgenomes in polyploid species during meiosis [134] and mitosis [135]. Centromeres are mainly composed of satellite repeats and centromeric retrotransposons (CR) [129,136,137]. It has been shown that both components are essential for centromere recognition by kinetochore proteins [127]. CR elements have been found in the centromeres of several plant genomes, such as rice [138], the coffee genus [15], brachypodium, wheat [139], maize [140], wild rice [10], and other cereals [141] and grasses [62,142]. Since CR elements contain a chromodomain region, they are probably able to interact with CENH3 proteins, suggesting their participation in the centromere function [15,143]. Given the high degree of repetitiveness of centromeric sequences [132], sequencing and assembly remain challenging, providing a partial view of the composition and organization of such regions in eukaryotes [131,135].

#### 2.3.2. Sex-Specific Chromosomes

Sex chromosomes are the portions of the genome that determine the sex of an individual. In flowering plants, some species show male and female flowers on separate individuals (dioecious species) [144,145], controlled genetically by specialized sex chromosomes. Sex chromosomes could originate from ancestral homologous chromosome pairs losing their potential to recombine. This suppression of recombination determines the sex-determining regions (SDR), and more generally induce a separate evolution of chromosomes [144,146], with the accumulation of TEs and other repetitive sequences and degenerating the gene content [146].

Sex chromosomes are known to accumulate repetitive sequences [80] due to suppression of recombination, but the sex-specific accumulation of transposable elements could also contribute to the differential repeat content of the X and Y chromosomes (the Y chromosome is larger than the X chromosome in *Silene latifolia*). This fact leads to size variation [147] in many reported genomes, such as sea buckthorn [148], papaya [149,150], *Silene latifolia*, *Coccinia grandis and Cannabis sativa* [124], as well as to other mechanisms that vary in dioecious species, such as population size and genome dynamics [148]. Further, TEs could be responsible for a lower gene content in the Y chromosome of *S. latifolia* (although the Y chromosome is the largest in this species and is ~1.4 times larger than the X chromosome [147]).

On the other hand, since plant Y chromosomes contain large non-recombining regions (and most of the species bear large Y chromosomes [147]), unequal homologous recombination between TEs can lead to large deletions. When the recombination involves long terminal repeats (LTRs) of the same retrotransposon, it results in the formation of solo-LTRs [149].

### 2.4. Interaction of Retrotransposons with Genes

One of the most interesting impacts that TEs could have on the host genomes and phenotypes [83] is the alteration of gene activity [52]. These impacts can include the imposition of intragenomic selection pressures through their effects on gene expression [76], inactivation of coding or regulatory regions of the gene [124], mutations that change the protein sequence, variation of the pattern of expression or alternative splicing [3], alteration of the expression of neighboring genes by epigenetic effects [82], or through modification of transcription factor expression [151], redirection of stress stimuli to adjacent genes [9], and the influence on the conservation, rearrangement, and deletion of gene pairs [152]. The long-term impact of such variation involves, for instance, genetic variation with important effects on species evolution [153], genomic diversification and speciation [154], and modification of the host fitness [89,108,155,156] by producing sense or antisense transcripts of the genes [88]. A known example of gene expression reprogramming is the one described by McClintock for anthocyanin pigment gene expression in maize [78] and wheat, where the activated retrotransposons Wis2-1A altered the expression of their adjacent genes [108]. Other methods to regulate gene expression occur at the transcriptional level through promoters and enhancers, which are well characterized in several retrotransposons [128,157], and at the post-transcriptional level through the production of microRNAs [3]. In addition, regulation could also take place by the silencing of some retrotransposons, which, in turn, silence adjacent genes in the opposite orientation [57], since the integration of a retrotransposon is generally accompanied by the methylation of the insertion region [153].

As with chromosomal distribution, retrotransposon families can be differently inserted into gene-rich regions [158]. For example, LTR-RTs commonly target their reinsertion to specific genomic sites around genes, promoting important putative functional implications for a host gene [29]. In barley, LINEs and SINEs were found more frequently at approximately 3 kb upstream of the transcription start site (TSS) and 5 kb downstream of genes, while the frequency of LTR-RTs decreased considerably. Additionally, SINEs were found nearly four times more frequently immediately up- and downstream of genes than at a distance of 10 kb, but LINEs were more frequent near genes [159]. 

LTR-RTs are also directly involved in gene creation and innovation [3] through transposon-based or retrotransposon-based gene capture [43] and domestication. More than 400 genes have been reported as LTR-RTs-captured genes in maize, 672 genes in rice, and 1343 in sorghum [140]. Several genes captured by non-LTR retrotransposons were designated as retrogenes [140]. The total number of genes domesticated through LTR-RTs is probably underestimated and should increase with the release of new genome sequences in the near future [81].

## 3. Why Is It Important to Classify Retrotransposons (into Superfamilies and Lineages)?

Since transposable elements constitute a substantial part of plant genomes (up to 85%) [81], their characterization and classification are necessary to understand the dynamics and mechanisms of genome evolution [40,52,124,160]. In addition, the annotation of TEs may improve the accuracy of coding region annotations and facilitate functional gene studies [53], relying on the development of different strategies of automatic bioinformatic identification and classification.

There is evidence that different families of retrotransposons may present different levels of activity [154,161] or represent different fractions of the genome [42,65]. For instance, it is well-known that the Gypsy and Copia superfamilies of LTR retrotransposons have considerable differences in their proportions of total genomic size [36]. Furthermore, retrotransposons can also display different evolutionary rates within a genus, as in the case of the *Coffea*, where the lineage Del (part of the Gypsy superfamily) shows an overall increase in the west from Indonesian and Malagasy *Coffea* species to East and West African species [162]. Finally, a given genomic region can harbor certain elements. For instance, centromeres contain a specific lineage of Gypsy retrotransposons [163].

### 3.1. Current Classifications

The first categorization of TEs was proposed by Finnegan in 1989, in which TEs are classified according to their intermediate molecules, DNA or RNA, and to the basic nature of their transposition mechanisms. Currently, the most used nomenclature was proposed by Wicker et al. [8], which also takes into account the transposition mechanism. However, due to the high diversity of TE structures and transposition mechanisms, there are still numerous classification problems and debates on classification systems [164,165].

**Figure 6:**
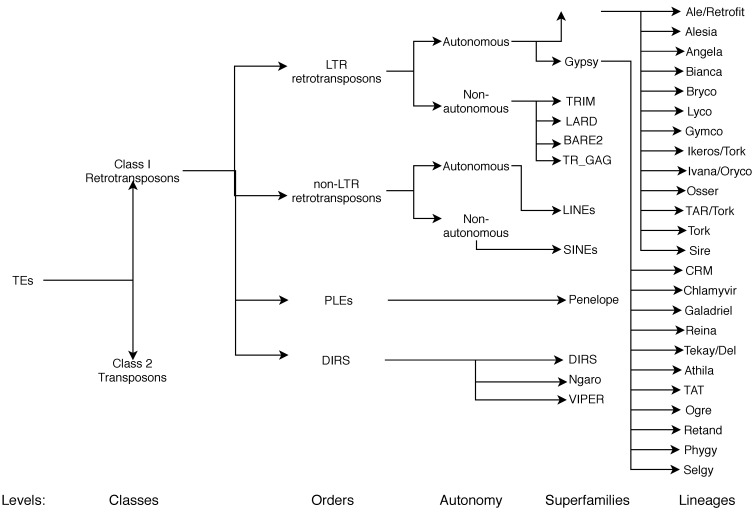
Classification of TEs following Rexdb and GyDB nomenclature. Adapted from [26].

In recent years, a considerable effort has been made to create a unified classification and nomenclature system. One of the most accepted methods was the hierarchical classification system that subdivided TEs into classes, subclasses, orders, superfamilies, lineages, and families, as proposed by Wicker et al. [8] (Figure 6).

As we mentioned earlier in this section, classification and nomenclature are still debated, and this is particularly true at the lineage level for LTR-RTs. On one hand, some authors proposed that the Copia superfamily was composed of AleI/Retrofit/Hopscotch, AleII, Angela, Bianca, Ivana/Oryco, Maximus/SIRE, and TAR/Tork; and Gypsy was composed of Athila, Chromovirus (which can be further classified into Reina, CRM, Galadriel, and Del [44]), and Ogre/TAT [39,52]. On the other hand, Llorens et al. proposed that *Copia* can be subdivided into Retrofit, Tork, Sire, and Oryco, and Gypsy into Athila, Tat, Reina, CRM, Galadriel, and Del [44]. Additionally, other studies group TAR, Ivana, Maximum, Ale, Bianca, and Angela into Copia and Tat, Athila, Reina, CRM, and Tekay into Gypsy [36]. Recently, Neumann et al. [16] introduced minor lineages (present in very few species) and subdivided Tork in the overall classification system (Figure 6). These different systems and their correspondence can be consulted in Table 3.

### 3.2. Current Nomenclature

Given the similarity of TEs with retroviruses and the huge diversity within orders, superfamilies, and lineages, it is common to find different names for the same subdivision, corresponding to different nomenclature systems (Table 3).

## 4. How to Identify and Classify Retrotransposons

Although the correct discovery of TEs is a crucial step in the annotation of newly sequenced genomes [168], the identification and classification (especially at the lower levels [33], i.e., lineage and family) of these elements is a very difficult task for almost all genomic projects [169] due to the wide diversity of structural features they present [121]. Because of the abundance of TEs of diverse classes and orders in the genomes (especially in species with huge genomes), the tasks of identification and classification are essential, not only for researchers who are interested in repeat composition, but also for those studying genome evolution, gene function, expression, and regulation of expression, among others [70,77]. Many bioinformatics software has been developed to detect and classify TEs, following varied methodologies and strategies with different accuracies [165,170] yet, in many cases, leaving large uncategorized and unexplored sections in sequenced plant genomes [171].

### 4.1. Current Problems for Retrotransposon Identification and Classification

Since TEs are under relatively low selection pressure and they evolve more rapidly than coding genes [172], these elements display a dynamic evolution due to insertions of other TEs into their sequences (nested insertion), illegitimate and unequal recombination, cellular gene capture, and inter-chromosomal and tandem duplications [173]. For this reason, their classification and further annotation is a very complex task [56]. Many attempts have been made to create a unified system of classification that combines both the phylogenetic and enzymatic aspects, yet, unfortunately, classification becomes more difficult at lower levels, such as superfamilies and lineages [33]. In some cases, complex research is required by specialists.

TEs with uniform structures and well-established mechanisms of transposition can be easily grouped and classified, such as for LTR retrotransposons [37]. However, in the case of no-autonomous elements, deletions, or groups with few shared features, the classification process remains challenging.

Besides natural diversity, most gene prediction programs tend to mix ORFs from many TEs with additional exons within genes, corrupting the final results [77], so TE identification and “masking” is highly recommended prior to annotation [77]. Finally, unlike gene annotation, the use of databases or reference sequences of TEs for identification or classification is a major challenge, because TEs are species-specific. Consequently, the TEs of most recently sequenced species are unknown [18].

The problems with the identification and classification of TEs are mainly:

- The difficulties in constructing a representative and comprehensive library of TE sequences, since it depends on the sensibility and specificity of the bioinformatics programs used.
- Nested elements.
- The false identification of TEs (for example, large gene families).
- The difficulties in classifying non-autonomous elements.

### 4.2. Current Strategies and Methodologies

There is no single tool that can be applied universally across all species for all TE types [165]. Therefore, many different techniques, methods, and software can be found in the literature. In this manner, there are diverse ways to group techniques or methods for identifying TEs. Most authors have proposed some of the following categories [26,140,170,172]: structure-based, homology-based, de novo, and comparative genomics. Further, many tools apply more than one method to improve their results [74].

#### 4.2.1. Structure-Based Methods

The algorithms following this approach search the presence of TEs according to a priori information about structural features [170,174]. These include duplications or duplicated inversion (LTR for LTR-RTs, TIR for most DNA transposons), short motifs such as TSDs, PPT, and PBS for LTR-RTs, and poly-A tails [77] for LINEs. These methods do not require libraries of known TEs or large repetitions of each TE in the genome. In this way, these methods can find elements with few copy numbers [77]. However, structure-based methods are not able to identify TEs with novel structures or elements that lack the main structural features.

#### 4.2.2. Homology-Based Methods

This strategy detects TEs on the basis of their similarity with reference TE sequences [121,175]. When a TE library or repeat database is available for the species studied, the identification process can be straightforward. The creation of a library for this method can be acquired in two ways: through existing databases (Table 5) or libraries constructed by de novo or other methods [169]. This can be achieved using any sequence alignment tool, such as BLAST, which will find TEs with a similarity value higher than a threshold [77], or with RepeatMasker [176]. The difficulties of this approach include the complexity in creating an accurate library of reference TEs, the huge diversity of these elements at the nucleotide level, and the species-specific characteristics of TEs. At the lineage and family levels, phylogenetic approaches (homology-based) are the most commonly used [53]. This method requires a library of known enzymatic domains categorized by lineages. Phylogenetic analyses are usually performed using RT domains, because these genes are the most conserved across species even though retrotransposons are highly variable in their sequences [115,177].

#### 4.2.3. De Novo

This approach looks for similar sequences found at multiple positions within a sequence [170] by taking advantage of the repetitive nature of TEs [18]. It can be executed in two ways: “self-comparison”, which requires aligning a genome, or sections of it, to itself. In this case, sensitivity depends on how significant aligned pairs are filtered [174]. The second way is through counting exact or approximate (known as “spaced”) k-mers [18,174]. This method is called de novo, because it does not require any additional information about the query sequences [77]. However, low-copy number TEs may not be recognized as repeated sequences in this approach.

#### 4.2.4. Comparative Genomics

In this strategy, whole genome sequences are compared to each other in order to identify indel regions caused by TEs [170]. The limitations of this approach include the need for a well annotated reference genome and the fact that TEs and special non-coding parts of TEs can show an enormous divergence between distantly and closely related species.

### 4.3. Most Popular Bioinformatics Resources

Much bioinformatics software has been developed following the aforementioned strategies, and most of them can only identify specific classes (retrotransposons or DNA transposons) or orders such as LTR-RTs or non-LTR retrotransposons (Table 2). Although data mining [6] and machine learning techniques have shown very successful results in other genomic tasks, very few tools for TEs apply these computational techniques in their algorithms (Table 4).

Interestingly, most of the software used to identify TEs requires assembled sequences as input, even though assembly algorithms have trouble with highly repetitive sections of genomes [4,66,203,204]. Repeats cause branches in graphs used in assembly algorithms (which can be one of two classes: overlap-based and De Bruijn graph) [205], leading assemblers to create false joins and wrong copy numbers, or even break graphs at these branch points, generating an accurate but fragmented assembly [205]. Indeed, sequences that are categorized as unknown or non-assembled in genomic projects are generally composed mainly by repetitive elements.

Additionally, many databases have been published in recent years, creating unique opportunities to compare thousands of TEs at all levels of classification from different plant species and taxa (Table 5).

## 5. How can Machine Learning and Deep Learning Techniques Improve the Identification and Classification of Retrotransposons?

Machine learning (ML) is a research area that aims to create algorithms that learn automatically. ML tasks can be divided into two categories: supervised and unsupervised. In supervised learning, the aim is to predict the label (classification) or response (regression) of each sample by using a provided set of training examples (prior classified data set). In unsupervised learning, such as clustering and principal component analysis (PCA), the goal is to learn inherent patterns within the data on its own [206]. Supervised learning algorithms are recommended when a high-quality data set is available to train the algorithms.

In general, the data set is divided into two or three subsets. The training set is used for learning the model, which can represent the calculation of several parameters depending on the algorithm used. The validation set is used to select the best model, and the test set is used to estimate the real performance of the model [206]. ML techniques have the ability to derive rules or features from the data without prior information [26]. For this reason, many bioinformatics researchers have used ML in their work.

One of the most important tasks in ML algorithms is correct data representation. In contrast to other data sets, DNA nucleotide sequences are recorded as human readable characters, C, T, A, and G. Thus, it is necessary to encode them in a machine-readable form [207]. Table 6 shows several coding schemes that can be applied to represent the nucleotides following different approaches.

On the other hand, deep learning (DL) has evolved as a sub-discipline of ML through the development of deep neural networks (DNN, i.e., neural networks with many hidden layers), such as auto-encoders, fully connected DNNs, convolutional neural networks, and recurrent neural networks, among others [208]. In DL, the issue of selecting the correct data representation or best features is included in the ML problem to yield end-to-end models [208]. DL has demonstrated very successful results in life sciences [207], especially in genomics, by identifying different types of genomic elements, like exons, introns, promoters, enhancers, positioned nucleosomes, splice sites, untranslated regions (UTR), etc. [157].

### 5.1. Current Machine Learning Techniques for Genomics and Transposable Elements

Techniques such as Support Vector Machines (SVMs), random forest, hidden Markov models (HMM), neural networks, and graphical models can be successfully applied to biological data because of their capabilities in handling randomness and the uncertainty of data noise, as well as their skill in generalization [215].

SVMs were applied to the classification process of TEs, such as in TEClass [168], and recently in the identification of Helitrons (an order of Class II transposons) [216], showing high precision rates. On the other hand, the TE-Learner framework uses a random forest to classify LTR retrotransposons, but the identification is done using traditional bioinformatics approaches [26]. Further, HMMs were used in RED software to identify repeats directly from sequencing reads [18]. One of the most important contributions of RED is the automatized label process that is done manually (in most cases). In addition, HMMs have been applied to aligning and constructing phylogenies using LTRs instead of the RT domain, since this technique allows noise removal from the data [63].

An additional novel method to identify mobile genetic elements was presented by Tsafnat and coworkers [217], in which the authors took advantage of the parallel between grammatical language recognition (which is a well-known ML problem) and the DNA language of life, by looking for element boundaries.

**Figure 7:**
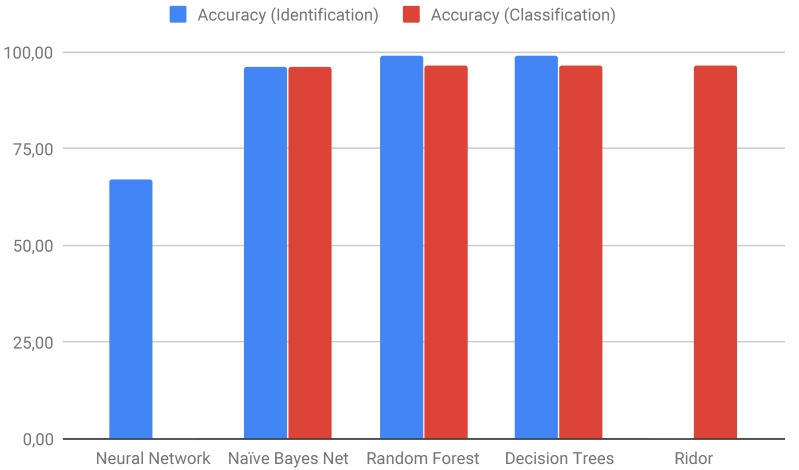
Accuracy of machine learning (ML) algorithms tested for TE identification and classification problems. A Neural Network and Ridor were used for only one problem. Adapted from Loureiro et al. [170].

Other ML techniques have been tested by Loureiro et al. [170] (Figure 7) for the detection and classification of TEs using results obtained by bioinformatics software such as Blat, Censor, LTR_finder, and RepeatMasker. They also used randomly generated sequences with different parameters to test different algorithms, as implemented in Weka (Table 7).

Recent works proposed a novel strategy to classify TEs using Hierarchical Classification (HC), since the classification system proposed by Wicker et al. [8] showed different levels of divisions, and this problem can be resolved by HC [218,219,220].

Although several studies have demonstrated the benefits of using ML in many biological problems, just a few software take advantage of this approach. Most algorithms available in literature use ML to address the classification problem, yet so far RED software uses ML to detect repeats but not to classify them.

### 5.2. Current Deep Neural Networks Techniques for Genomics and Transposable Elements

Recent advances in ML have proven that DNN can obtain better results than common neural networks. Additionally, DL techniques like auto-encoders (AE), denoising auto-encoders (DAE), and their stacked versions have expanded to state-of-the-art fields of study, including bioinformatics [220].

Regarding the TE problems discussed in this review, DL has been applied to classification using HC, suggesting that employing DNNs with an increasing number of hidden layers can yield slightly better results, excelling methods in the literature [220].

On the other hand, auto-encoders have been used to detect long intergenic non-coding RNA (lincRNA), showing very interesting results [207] and improving the results from SVM. Considering that TEs are composed of long non-coding regions, the techniques used in the latter research could be used on the TE problems addressed in this article.

Although the intersection of DL methods and genomic research may lead to a profound understanding of genomics [157], so far, no software was found to use DL for the identification and classification of TEs. Also, there is a large bibliography on the use of DL in other areas of genomics (reviewed in [157]), including functional genomics, gene expression, regulatory genomics, among others, suggesting that the application of DL to TE problems can be useful to overcome difficulties.

## 6. Conclusions

Initially considered “junk DNA” [81], transposable elements became a gold mine for evolutionary genomics researchers studying genome evolution and adaptation, as well as for those studying new strategies to increase crop genome diversity. Indeed, the advances of next generation sequencing (NGS) technologies revolutionized biology and provided new opportunities to study very huge and complex genomes, such as maize or sugarcane. However, NGS is also a challenge for bioinformatics algorithms. How do we identify and classify transposable elements in thousands of genome sequences [221] in a reasonable time? New and efficient bioinformatics algorithms are highly required to transition between the analyses of dozens to thousands of genomes. ML and DL may represent the new generation of bioinformatics approaches, especially for TEs [214]. Both techniques have been tested in many genomic areas, demonstrating very high levels of success, yet their application in TEs is limited. Currently, new algorithms applying ML or DL and traditional techniques must be developed in order to overcome the problems of TEs and simplify the assembly and annotation processes in future genomics. Using key features like retrotransposon length, LTR length, ORFs, and motifs such as the TATA box, AATAAA, TDS, and poly-A tails, one it seems possible to build a well-defined ML problem. Using data mining, Arango-López et al. (2017) [6] demonstrated that element length and LTR length are important to classify LTR retrotransposons, Benachenhou et al. [64] proposed that motifs inside of LTRs are conserved across superfamilies using HMMs, Fischer et al. (2018) [222] showed that profile hidden Markov models (pHMMs) are a promising approach to find TEs in genomes, and Orozco-Arias et al. (2017) [223] demonstrated the useful of high performance computing to speed up analysis of TEs in large genomes. Finally, Loureiro et al. [170] presented evidence that ML can be used to test and improve the identification and classification of TEs using already developed bioinformatics tools. In addition to already-tested ML algorithms and techniques in TE problems, the availability of many databases with thousands of TEs provides an opportunity to apply ML, because the training process can be improved using a large amount of previously classified TEs, with the aim to obtain a more general and optimal model. Nevertheless, ML and DL cannot solve all of the problems in the identification and classification of TEs. One challenge in the field will be to build comprehensive software integrating a combination of different approaches of TE detection to perform accurate genome annotation.

## Figures and Tables

**Table 1:** Transposable element domains and their function in the replication mechanism. Adapted from [6,55]. LTR, long terminal repeat.

Complete Gene Name	Short Name	Function
*Reverse transcriptase*	*RT*	Responsible for DNA synthesis using RNA as a template
*RNase H*	*RNAseH*	Responsible for the degradation of the RNA template in the DNA-RNA hybrid
*Intregrase*	*INT*	Responsible for catalyzing the insertion of the retrotransposon cDNA into the genome of a host cell
*Aspartic protease*	*AP*	Responsible for processing large transposon transcripts into smaller protein products
*Envelope*	*ENV*	Responsible for cell-to-cell transfer of retroviruses.
*Group specific antigen*	*GAG*	Structural protein for virus-like particles
*Chromodomain*	*Chrod*	Responsible for targeting the insertion of new LTR retrotransposon copies into heterochromatic regions by recognizing specific heterochromatic histone marks and/or other factors

**Table 2:** Stress-activated retrotransposons reported in plant genomes. With information from [9,34,84,86,90,91].

Retrotransposon	Stresses by External Conditions	Species	Reference
*Tnt1*	Protoplast and tissue culture, pathogens, pathogen elicitors, compounds related to plant defense, wounding, freezing, in vitro regeneration, mechanical damage, and microbial factors.	Tobacco	[92]
*Tto1*	Wounding, methyl jasmonate, tissue culture, fungal elicitors, chilling, cytosine demethylation, resistance to bacterial blight, and plant development.	Tobacco	[93]
*Tos17*	Tissue culture and viral infection.	Rice	[94]
*OARE-1*	Wounding, jasmonic and salicylic acid, UV light, infection with an incompatible race of the crown rust fungus.	Oat	[95]
*Reme1*	UV light.	Melon	[96]
*ONSEN*	Heat stress.	*A. thaliana* and other members of the Brassicaceae family	[97]
*GBRE-1*	Heat stress.	*Gossypium*	[98]
*FaRE1*	Hormonal treatments.	Strawberry	[99]
*BARE-1*	Water-induced stress.	Barley, *Hordeum spontaneum*	[100]
*Tlc1*	Phytohormones, wounding, protoplast preparation, high salt concentration and stress-associated signaling molecules.	*Solanum chilense*	[101]
*Erika*	Fungal infection.	Wild wheat	[57]
*Bs1*	Barley stripe mosaic virus infection.	Maize	[102]
*ZmMI1*	Cold.	Maize	[103]
*CLCoi1*	Wounding and salt stress.	Lemon	[90]
*MAGGY*	Heat shock.	Rice	[104]
*Wis2-1A*	Interspecific hybridization.	Wheat	[57]
*LORE1*	Tissue culture.	*Lotus japonicus*	[105]

**Table 3:** Correspondences between names of superfamilies and lineages given for some classification systems and the International Committee on Taxonomy of Viruses (ICTV). Adapted from [16,37,63].

Superfamilies			
REXdb ^a^	Wicker and Keller ^b^	GyDB ^c^	ICTV ^d^
Copia	Copia	Ty1/Copia	Pseudoviridae
Gypsy	Gypsy	Ty3/Gypsy	Metaviridae
Bel-pao	Bel-pao	Bel-pao	Semotiviruses
Lineages (Copia)			
Ale	Ale	Sirevirus/Retrofit	pseudovirus
Alesia	Ale	-	-
Angela	Angela	-	pseudovirus
Bianca	Bianca	-	-
Bryco	-	-	-
Lyco	-	-	-
Gymco-I, II, III, IV	-	-	-
Ikeros	Angela	Tork	pseudovirus
Ivana	Ivana	Sirevirus/Oryco	-
Osser	-	Osser	hemivirus
SIRE	Maximus	Sirevirus/SIRE	Sirevirus
TAR	TAR	Tork	-
Tork	-	Tork	pseudovirus
Lineages (Gypsy)			
chromovirus|CRM	-	chromoviruses|CRM	-
chromovirus|Chlamyvir	-	-	-
chromovirus|Galadriel	-	chromoviruses|Galadriel	-
chromovirus|Reina	-	chromoviruses|Reina	-
chromovirus|Tekay	-	chromoviruses|Del	Metavirus (Del1)
non-chromovirus|OTA|Athila	-	Athila/Tat|Athila	Metavirus (Athila)
non-chromovirus|OTA|Tat|TatI	-	-	-
non-chromovirus|OTA|Tat|TatII	-	-	-
non-chromovirus|OTA|Tat|TatIII	-	-	-
non-chromovirus|OTA|Tat|Ogre	-	Athila/Tat|Tat (Ogre)	-
non-chromovirus|OTA|Tat|Retand	-	Athila/Tat|Tat	Metavirus (Tat4)
non-chromovirus|Phygy	-	-	-
non-chromovirus|Selgy	-	-	-

^a^ [16], ^b^ [166], ^c^ [14], ^d^ [167].

**Table 4:** Bioinformatics software found in the literature. I for identification, C for classification, and O for other analysis; ML for machine learning. With information from [18,29,77].

Software	Approach	TE Class or Order	Applies ML	Input Format Files	Tasks	Reference
Censor	Homology-based	Any	NO	Any	I	[178]
Find_ltr	Structure-based, Homology-based	Complete LTR RTs, and solo LTRs	NO	Assembled sequences	I	[179]
FORRepeats	Homology-based	Any	NO	Any	I	[180]
Inpactor	Structure-based, Homology-based	LTR RTs	NO	Assembled sequences, LTR_Struc output or REPET output	C, O	[23]
LTR-FINDER	Structure-based	LTR RTs	NO	Assembled sequences	I	[181]
LTR_MINER	Structure-based	LTR RTs	NO	RepeatMasker output	I	[182]
LTR_retriever	Structure-based	LTR RTs	NO	Assembled sequences	I	[183]
LTR_STRUC	Structure-based	LTR RTs	NO	Assembled sequences	I	[184]
LTRClassifier	Homology-based	LTR RTs	NO	Assembled sequences	C	[22]
LTRdigest	Structure-based, Homology-based	LTR RTs	NO	LTRharvest output	C	[185]
LTRHarvest	Structure-based	LTR RTs	NO	Assembled sequences	I	[186]
LTRSift	Structure-based	LTR RTs	NO	LTRdigest output	C	[187]
LTRType	Homology-based	LTR RTs	NO	Assembled sequences	I	[188]
P-Clouds	De novo	Any	NO	Assembled sequences	I	[189]
PASTEC	Structure-based, Homology-based	Any	NO	Assembled sequences	C	[190]
PILER	Structure-based, De novo	Any	NO	Assembled sequences	I	[191]
RAP	De novo	Any	NO	Assembled sequences	I	[192]
REannotate	Other	Any	NO	RepeatMasker output	O	[193]
ReAS	De novo	Any	NO	Unassembled sequence reads	I	[194]
RECON	De novo	Any	NO	Unassembled and assembled sequences	I	[195]
Red	De novo (HMM)	Any	YES	Unassembled and assembled sequences	I	[18]
REDdenovo	De novo	Any	NO	Unassembled sequence reads	I	[21]
REPCLASS	Structure-based, Homology-based	Any	NO	Assembled sequences	I	[196]
RepeatExplorer	De novo	Any	NO	Unassembled sequence reads	I	[197]
RepeatMasker	Homology-based	Any	NO	Assembled sequences	O	http://www.repeatmasker.org/
RepeatModeler	De novo	Any	NO	Assembled sequences	I	http://www.repeatmasker.org/RepeatModeler/
RepeatScout	De novo	Any	NO	Assembled sequences	I	[198]
Repeat Pattern	De novo	Any	NO	Assembled sequences	I	[199]
REPET	De novo, Structure-based,	Any	NO	Assembled sequences	I, C	[200]
Repseek	De novo	Any	NO	Assembled sequences	I	[201]
REPuter	De novo	Any	NO	Assembled sequences	I	[202]
TEClass	De novo (SVM)	Any	YES	Assembled sequences	C	[17]
TEdna	De novo	Any	NO	Unassembled sequence reads	I	[19]
transposome	De novo	Any	NO	Unassembled sequence reads	I	[20]

**Table 5:** TE databases available.

Database	Genomes	Data Composition	URL
Gypsy database	Several plant genomes	Domains from LTR Retrotransposons	http://gydb.org/index.php/Main_Page
MASiVEdb	Several plant genomes	Sire Retrotransposons	http://databases.bat.ina.certh.gr/masivedb/
Repbase	Several plant genomes	All TEs	https://www.girinst.org/repbase/
RepPop	*Populus trichocarpa*	All TEs	http://csbl.bmb.uga.edu/~ffzhou/RepPop/
RetrOryza	Rice	LTR Retrotransposons	http://retroryza.fr
REXdb	Several plant genomes	Domains form LTR Retrotransposons	http://repeatexplorer.org/?page_id=918
SINEBase	Several plant genomes	SINEs	http://sines.eimb.ru/
SoyTEdb	Soybean	All TEs	https://soybase.org/soytedb/
TIGR Maize repeat database	Maize	All TEs	http://maize.jcvi.org/repeat_db.shtml
TRansposable Elements Platform (TREP) database	Several cereal genomes	All TEs	http://botserv2.uzh.ch/kelldata/trep-db/
Plant Genome and System Biology (PGSB) Repeat Database	Several plant genomes	All TEs	http://pgsb.helmholtz-muenchen.de/plant/recat/
RepetDB	Several plant genomes	All TE consensus	http://urgi.versailles.inra.fr/repetdb/begin.do

**Table 6:** Coding schemes for the translation of DNA characters into numerical representations. Adapted from [209].

Coding Schemes	Codebook	Reference
DAX	{‘C’:0, ‘T’:1, ‘A’:2, ‘G’:3}	[210]
EIIP	{‘C’:0.1340, ‘T’:0.1335, ‘A’:0.1260, ‘G’:0.0806}	[211]
Complementary	{‘C’:-1, ‘T’:-2, ‘A’:2, ‘G’:1}	[212]
Enthalpy	{‘CC’:0.11, ‘TT’:0.091, ‘AA’:0.091, ‘GG’:0.11, ‘CT’:0.078, ‘TA’:0.06, ‘AG’:0.078, ‘CA’:0.058, ‘TG’:0.058, ‘CG’: 0.119, ‘TC’:0.056, ‘AT’:0.086, ‘GA’:0.056, ‘AC’:0.065, ‘GT’:0.065, ‘GC’:0.1111}	[213]
Galois (4)	{‘CC’:0.0, ‘CT’:1.0, ‘CA’:2.0, ‘CG’:3.0, ‘TC’:4.0, ‘TT’:5.0, ‘TA’:6.0, ‘TG’:7.0, ‘AC:8.0, ‘AT: 9.0, ‘AA’:1.0, ‘AG:11.0, ‘GC’:12.0, ‘GT’:13.0, ‘GA’:14.0, ‘GG’:15.0 }	[209]
Orthogonal Encoding	{‘A’: [1, 0, 0, 0], ‘C’: [0, 1, 0, 0], ‘T’: [0, 0, 1, 0], ‘G’: [0, 0, 0, 1]}	[214]

**Table 7:** ML algorithms tested by Loureiro et al. [170] in TE identification and classification problems.

Identification		Classification	
Algorithm	Accuracy	Algorithm	Accuracy
Neural Network	67.01	Ridor	96.43
Naïve Bayes Net	96.30	Naïve Bayes Net	96.37
Random Forest	98.90	Random Forest	96.56
Decision Trees	98.92	Decision Trees	96.56

## Abbreviations

AP	Aspartic protease
CR	centromeric retrotransposons
DIRS	Dictyostelium intermediate repeat sequence
DL	Deep Learning
DNN	Deep Neural Networks
ENV	Enveloppe
FISH	Fluorescent In Situ Hybridization
GAG	Group Specific Antigen
HC	Hierarchical Classification
HMM	Hidden Markov Models
HT	Horizontal Transfer
HTT	Horizontal Transfer of Transposable element
indel	Insersion-Deletion
INT	Integrase
LINE	Long Interspersed Nuclear Element
LTR	Long Terminal Repeat
LTR-RT	Long Terminal Repeat retrotransposon
ML	Machine learning
NGS	Next Generation Sequencing
ORF	Open Reading Frame
PBS	primer binding site
PPT	Poly-Purine Tract
PLEs	Penelope-like elements
RT	Reverse transcriptase
SINE	Short Interspersed Nuclear Element
SVM	Support Vector Machine
TE	transposable elements
TIR	Terminal Inverted Repeat
TSD	Target Site Duplication
UTR	Untranslated Regions
